# Age differences in physiological responses to self-paced and incremental $\dot{V}{\text{O}}_{2\hbox{max} }$ testing

**DOI:** 10.1007/s00421-016-3508-6

**Published:** 2016-12-10

**Authors:** Lauren A. Jenkins, Alexis R. Mauger, James G. Hopker

**Affiliations:** STRAPH Research Group, School of Sport and Exercise Sciences, University of Kent, Chatham Maritime, Kent, UK

**Keywords:** RPE, Maximal exercise, Cardiac output, Pacing

## Abstract

**Purpose:**

A self-paced maximal exercise protocol has demonstrated higher $\dot{V}{\text{O}}_{2\hbox{max} }$ values when compared against traditional tests. The aim was to compare physiological responses to this self-paced $\dot{V}{\text{O}}_{2\hbox{max} }$ protocol (SPV) in comparison to a traditional ramp $\dot{V}{\text{O}}_{2\hbox{max} }$ (RAMP) protocol in young (18–30 years) and old (50–75 years) participants.

**Methods:**

Forty-four participants (22 young; 22 old) completed both protocols in a randomised, counter-balanced, crossover design. The SPV included 5 × 2 min stages, participants were able to self-regulate their power output (PO) by using incremental ‘clamps’ in ratings of perceived exertion. The RAMP consisted of either 15 or 20 W min^−1^.

**Results:**

Expired gases, cardiac output (*Q*), stroke volume (SV), muscular deoxyhaemoglobin (deoxyHb) and electromyography (EMG) at the vastus lateralis were recorded throughout. Results demonstrated significantly higher $\dot{V}{\text{O}}_{2\hbox{max} }$ in the SPV (49.68 ± 10.26 ml kg^−1^ min^−1^) vs. the RAMP (47.70 ± 9.98 ml kg^−1^ min^−1^) in the young, but not in the old group (>0.05). *Q* and SV were significantly higher in the SPV vs. the RAMP in the young (<0.05) but not in the old group (>0.05). No differences seen in deoxyHb and EMG for either age groups (>0.05). Peak PO was significantly higher in the SPV vs. the RAMP in both age groups (<0.05).

**Conclusion:**

Findings demonstrate that the SPV produces higher $\dot{V}{\text{O}}_{2\hbox{max} }$, peak *Q* and SV values in the young group. However, older participants achieved similar $\dot{V}{\text{O}}_{2\hbox{max} }$ values in both protocols, mostly likely due to age-related differences in cardiovascular responses to incremental exercise, despite them achieving a higher physiological workload in the SPV.

## Introduction

A self-paced maximal oxygen uptake ($\dot{V}{\text{O}}_{2\hbox{max} }$) test (SPV) allows participants to regulate their own exercise intensity according to specific Ratings of Perceived Exertion (RPE), which follows an incremental format over 5 × 2 min stages (11–20 RPE). The SPV has been shown to allow young participants to achieve a higher $\dot{V}{\text{O}}_{2\hbox{max} }$ when compared to a standard incremental exercise test in both cycling and running exercise modes (Astorino et al. 2015; Mauger and Sculthorpe 2012; Mauger et al. 2013), although a mechanistic explanation is yet to be identified. However, not all studies have found differences in $\dot{V}{\text{O}}_{2\hbox{max} }$ when comparing the SPV vs. standard incremental exercise protocols (Chidnok et al. 2013; Faulkner et al. 2015; Straub et al. 2014).

Maximal oxygen consumption is suggested to be limited by factors such as: pulmonary diffusion capacity, maximal cardiac output, oxygen carrying capacity of the blood, or skeletal muscle characteristics (Basset and Howley 2000). Mauger et al. (2013) suggested that a higher $\dot{V}{\text{O}}_{2\hbox{max} }$ may be achieved during an SPV protocol due to changes in oxygen extraction at the working muscles, rather than any increase in oxygen delivery. The authors based this hypothesis on the observation that participants achieved a lower peak heart rate (HR) in the SPV without differences in peak minute ventilation (VE); they suggested that if the higher $\dot{V}{\text{O}}_{2\hbox{max} }$ were a result of an increase in the oxygen delivery it would be expected that both HR and VE would be increased (Mauger and Sculthorpe 2012; Mauger et al. 2013). However, it is possible that increases in stroke volume (SV), which was not measured by Mauger and Sculthorpe (2012) and Mauger et al. (2013), may have compensated for the lower HR achieved resulting in an increase in oxygen delivery, and thus $\dot{V}{\text{O}}_{2\hbox{max} }$ (Basset and Howley 2000). Nevertheless an increased oxygen extraction in the SPV may still arise from the participant being able to adjust work rate, potentially creating optimal conditions that allow enhanced rates of muscle oxygen extraction and higher work rates to be achieved. Indeed, previous research demonstrates that muscle blood flow, and thus oxygen extraction, is reduced when both muscle force and duration of contractions increase (Bjorklund et al. 2010; Hoelting et al. 2001). Research has also suggested that decreased blood transit time is associated with reduced rates of oxygen extraction (Basset and Howley 2000; Kalliokoski et al. 2001). In traditional incremental tests, the continuous increase in force requirement and consistent duration of each contraction cannot be adjusted. As a consequence this may result in a constriction of the amount of blood flow available to the muscle, and reduced time for extraction to take place. Both force and duration of muscle contractions are free for the participant to vary during the SPV, potentially optimising working muscle blood flow. In addition, it has been suggested that the self-regulation of work rate during the SPV may improve the efficiency of muscle recruitment, i.e. affording greater reliance on more oxygen efficient muscle fibres (Type I), particularly in the earlier stages of the test (Mauger and Sculthorpe 2012; Mauger et al. 2013). This self-regulation may help to conserve Type II fibres for the “all-out” final stage of the SPV protocol (RPE 20) which ultimately allows the high work rates to be achieved.

A recent study has demonstrated that the SPV may elicit a greater cardiac output (*Q*) and peak HR when compared to a standard incremental ramp protocol (Astorino et al. 2015). The study by Astorino et al. (2015) was the first to assess *Q* across the SPV, and suggests that the greater $\dot{V}{\text{O}}_{2\hbox{max} }$ may be explained by an increase in oxygen delivery (Mortensen et al. 2005), rather than an increase in the oxygen extraction as previously suggested. Astorino and colleagues (2015) suggested the increase in *Q* during the SPV protocol is likely the result of participants adequately pacing their effort to minimise fatigue in the early stages of the test. Ultimately, this better pacing led to a greater work rate being achieved in the final stage of the SPV, potentially because participants preserved the use of type II fibres in the earlier stages, resulting in a greater HR, *Q* and $\dot{V}{\text{O}}_{2\hbox{max} }$.

It is well accepted that there is a decline in $\dot{V}{\text{O}}_{2\hbox{max} }$ with aging, which is predominantly the result of reductions in maximal *Q* and muscle blood flow (Betik and Hepple 2008). Cardiac function (Lakatta and Levy 2003), lung performance (Chaunchaiyakul et al. 2004; Janssens et al. 1999) and muscle oxidative capacity (Betik and Hepple 2008; Russ and Kent-Braun 2004) have all been shown to reduce with age, which also contribute to the deterioration in $\dot{V}{\text{O}}_{2\hbox{max} }$. Therefore, the factors limiting factors $\dot{V}{\text{O}}_{2\hbox{max} }$ may differ between young and old populations. Consequently, selecting a $\dot{V}{\text{O}}_{2\hbox{max} }$ test protocol which adequately stresses the body’s physiological systems is an important decision when testing young and older populations. With the study of Astorino et al. (2015) demonstrating that the SPV protocol produces higher *Q* and $\dot{V}{\text{O}}_{2\hbox{max} }$ values compared to traditional methods, it could be speculated that a self-paced exercise test provides the best method to maximally stress the cardiorespiratory system. Furthermore, examining cardiopulmonary and muscular responses of young and old populations to both SPV and traditional test protocols may help explain why higher $\dot{V}{\text{O}}_{2\hbox{max} }$ values are often seen from the SPV. Therefore, the aim of the current study was to assess physiological responses to both SPV and standard incremental ramp test (RAMP) protocols in healthy younger (18–30) and older (50–75) populations, and to objectively test whether responses differ between the two groups.

## Methods

### Ethical approval

The study was conducted following institutional ethical approval in accordance with the Declaration of Helsinki (2013). All participants who volunteered gave their written informed consent.

### Participants

Forty-four healthy male and female participants, comprising of twenty-two 18–30-year-olds (age 25 ± 4 years; height 174 ± 11 cm; weight 69 ± 9 kg) and twenty-two 50–75-year-olds (age 59 ± 6 years; height 171 ± 8 cm; weight 73 ± 13 kg), volunteered to take part in the current study. All participants were apparently healthy, free of disease, free of any risk factors associated with cardiovascular disease and were all physically active (>90 min of moderate activity per week). This information was obtained via a health questionnaire. For the older population a resting blood pressure measurement was taken on their first visit to ensure they were not hypertensive.

### Experimental procedure

Each participant visited the laboratory on two separate occasions, where they were asked to complete either an SPV, or a traditional ramp $\dot{V}{\text{O}}_{2\hbox{max} }$ (RAMP) test using a randomised, counter-balanced, and crossover design. Tests were separated by at least 24 h to allow full recovery and were completed at the same time of the day (±2 h). Participants were asked to refrain from drinking alcohol (24 h abstinence), eating (2 h abstinence), and not to perform any exercise in the 24 h prior to each test. Prior to each test participants were required to complete a 5 min warm-up at approximately 50 W. In the SPV condition during the warm-up, participants were familiarised with the process of freely adjusting their power output (PO).

#### SPV protocol

The SPV was completed on an air-braked cycle ergometer (Wattbike Ltd, Nottingham, UK), which allowed participants to continually vary their PO throughout the test. The SPV was conducted in accordance with the procedures previously outlined by Mauger and Sculthorpe (2012). Recent data have demonstrated good test–retest reliability of both maximal and submaximal physiological data obtained from the SPV protocol in both young healthy, and older clinical populations (Jenkins et al. 2016). Briefly, the SPV consisted of 5 × 2-min stages (total test time of 10-min), where for each stage participants were able to continuously vary their PO, but with RPE (Borg 1998; 6–20 scale) fixed at a particular rating for each stage (RPE 11, 13, 15, 17 and 20), following an incremental format. Changes in PO were facilitated by the participants manually adjusting the cycle ergometer air brake and cadence in order to produce a work rate that allowed them to match the target RPE for each stage of the SPV. Participants were able to view their cadence and PO throughout the test; they also received feedback on elapsed time particularly when approaching the end of a stage.

#### RAMP protocol

The traditional $\dot{V}{\text{O}}_{2\hbox{max} }$ protocol was completed on an electro-magnetically braked cycle ergometer (Corival, Lode, Groningen, Netherlands) so that PO was fixed for each stage of the incremental RAMP protocol. The cycle ergometer was set in hyperbolic mode to ensure that any changes in cadence did not influence PO. Participants were instructed to keep their cadence above 60 rev min^−1^. The RAMP commenced with a 3 min period of baseline cycling where a various range of work rate starting points were selected for each participant (20, 50 and 100 W), according to self-reported fitness levels. This 3 min period was then followed by RAMP increments of either 15 or 20 W min^−1^. The starting point and the rate of increase in work rate was individualised in the attempt to optimise the protocol for each participant. The test was terminated when the participant could no longer continue, or if they were unable to maintain a cadence of more than 60 rev min^−1^, despite verbal encouragement.

### Physiological measures

#### Expired gases

During both exercise tests, expired gases were measured via the use of an online breath-by-breath analysis system (Cortex Metalyzer 3BR2, Cortex, Leipzig, Germany). After each test, $\dot{V}{\text{O}}_{2\hbox{max} }$ was calculated as the highest 30 s average $\dot{V}{\text{O}}_{2}$ (l min^−1^ and ml kg^−1^ min^−1^). Peak cycling PO and minute ventilation ($\dot{V}{\text{E}}$) were also both calculated as the highest 30 s average value. The anaerobic threshold (AT) was determined using the V-slope method with confirmation via the ventilatory equivalents ($\dot{V}{\text{E}}$/$\dot{V}{\text{O}}_{2}$ and $\dot{V}{\text{E}}$/$\dot{V}{\text{CO}}_{2}$) and the partial end-tidal (P_ET_O_2_ and P_ET_CO_2_) methods (Hopker et al. 2011). All AT’s were independently assessed by two experienced researchers. If the two researchers disagreed on the location of the AT, a third researcher was consulted.

#### NIRS

Muscle deoxyhaemoglobin (deoxyHb) was measured using a Near-Infrared Spectroscopy (NIRS) device (Portamon, Artinis Medical Systems, Elst, Netherlands). The device uses small skin surface lasers to measure light absorbance, operating at wavelengths between 760 and 850 nm, with an average optode distance of 35 mm and a sampling rate of 10 Hz. The device was placed longitudinally on the vastus lateralis (VL) of the left leg, and was situated 10 cm above the patella (Wang et al. 2012). Before placement of the NIRS device, the NIRS system was calibrated and the skin was carefully shaved. The device was secured to the site using adhesive tape, which covered the whole device to reduce light loss. Afterwards, the deoxyHb was exported into 1 s values and then averaged into 30 s at 10 time points over both tests. A baseline deoxyHb from the NIRS was averaged from the final 30 s of the first stage, and the peak was determined from the average of the final 15 s of each test. The deoxyHb for each time point was then normalized to the total amplitude of response (peak − baseline) (Boone et al. 2010). This was then plotted as a function of percentage of time to exhaustion (%TTE).

#### Electromyography (EMG)

Surface EMG was recorded using a wireless Biopac MP150 (Biopac Systems Inc, CA, USA), two surface electrodes were placed on the VL of the right leg and a reference electrode was placed on the patella of the same leg. The skin was prepared by carefully shaving and cleaning the area. EMG was recorded at a sampling frequency of 1000 Hz. Prior to each $\dot{V}{\text{O}}_{2\hbox{max} }$ test, participants performed three maximal isometric voluntary contractions (MIVC) of the VL muscle in order to determine the peak EMG signal during a maximal muscle contraction. The MIVCs were completed on a Cybex Isokinetic Dynamometer (HUMAC Norm, CSMi, Stoughton, MA, USA). Before the MIVCs a series of submaximal contractions were completed as a preparatory warm-up. Each MIVC lasted 5 s, with 1 min rest between each. Participants were also required to complete one MIVC directly after both $\dot{V}{\text{O}}_{2\hbox{max} }$ tests to determine the level of muscle fatigue, this occurred 1 min after the test or as close to 1 min as possible; depending on how quickly the participant was safely able to move off the bike and onto the dynamometer. Maximum EMG was calculated by averaging the highest 1 s EMG value from each MIVC trial. The 30 s average EMG signals from each stage of the SPV and RAMP was then normalized to the maximum EMG from the MIVC. Data were plotted as a function of %TTE from each $\dot{V}{\text{O}}_{2\hbox{max} }$ test. It was decided to include the whole 30 s of the EMG signal within the average, thus the inactive periods were included. The authors are aware that including the inactive periods between each contraction may been seen as a limitation as variations in cadence would influence the average of EMG (e.g. higher cadence would results in a higher EMG average). However, given the nature of the current study it was felt that excluding the inactive periods would not be necessary in order to provide an estimate of the overall muscle activity during each stage of the SPV or RAMP test.

#### Cardiac output and stroke volume

A non-invasive thoracic impedance device (PhysioFlow, Manatec Biomedical, France) was used to measure stoke volume (SV) and *Q* throughout the duration of both $\dot{V}{\text{O}}_{2\hbox{max} }$ tests. Electrodes were positioned in the following areas; above the supraclavicular fossa (participants’ left side), xiphoid process and two additional electrodes were placed to determine a single ECG signal at V1 and V6 positions. Prior to electrode placement, all skin sites were carefully cleaned, and shaved where necessary. In accordance with the manufacturer recommendations, the equipment was auto-calibrated prior to each test by establishing impedance waveforms over 30 heart beats. Peak *Q* and SV were determined by the highest 30 s average value over the entire duration of the test. A 30 s average of SV and *Q* was plotted, for every 2 min, as a function of percentage of time to exhaustion (%TTE) from each $\dot{V}{\text{O}}_{2\hbox{max} }$ test. Arteriovenous oxygen difference (a-vO_2_diff) was calculated as $\dot{V}{\text{O}}_{2\hbox{max} }$ (ml min^−1^)/*Q* (l min^−1^), and expressed in ml 100 ml^−1^. The *Q* value was taken as the value at $\dot{V}{\text{O}}_{2\hbox{max} }$, rather than peak *Q* achieved during the incremental test protocol.

#### Blood lactate

A finger-tip capillary blood sample was taken directly at the end of each $\dot{V}{\text{O}}_{2\hbox{max} }$ test to determine end-exercise blood lactate concentrations (Biosen C-Line, EKF Diagnostic, London, UK).

### Statistical analysis

All statistical analysis was conducted using SPSS (SPSS, Chicago, IL, USA) with significance levels accepted at 95% (*p* < 0.05). All data were checked for normality using the Shapiro–Wilk test and *Q*–*Q* plots. The mean maximum values for $\dot{V}{\text{O}}_{2\hbox{max} }$, AT, PO, HR, $\dot{V}{\text{E}}$, blood lactate, *Q* and SV were all compared between the protocols using a paired sample *t* test. The percentage difference between the pre-MIVC and post-MIVC (highest 1 s EMG and torque) from the SPV and RAMP was also compared using a paired sample *t* test. Differences (*p* < 0.05) between *Q*, SV, deoxyHb and EMG for the last 30 s of each stage, in both the SPV and RAMP tests were assessed using a repeated measures ANOVA (2 × 10 for NIRS and EMG; 2 × 6 for *Q* and SV). Violations of the assumptions were assessed using the Mauchly’s test of sphericity, if *p* was >0.05 then sphericity was assumed but if *p* was <0.05 then Greenhouse–Geisser corrections were used. If an interaction was present then Bonferroni post hoc testing was used to identify where the interaction occurred. The two defined groups, young (18–30 years) and old (50–75 years), were analysed separately. All data are presented as mean ± SD unless otherwise stated.

## Results

**Fig. 1:**
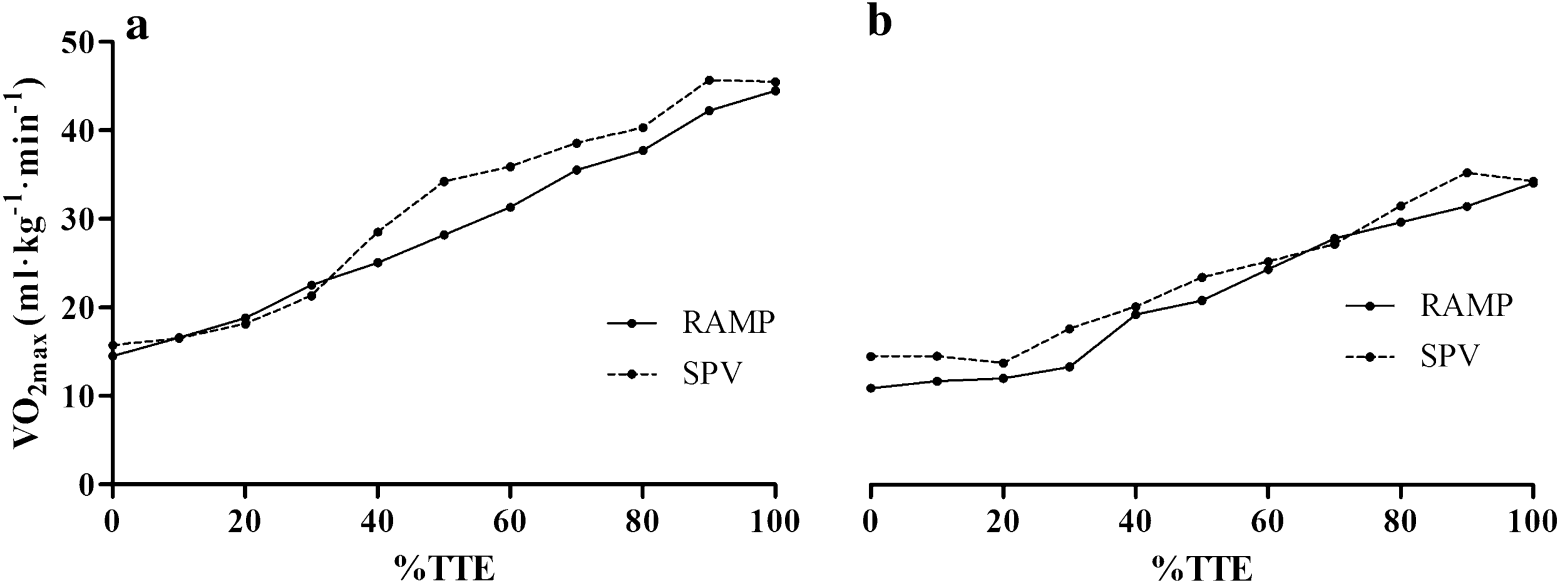
$\dot{V}{\text{O}}_{2}$ response over %TTE in the RAMP and SPV in a representative: **a** young and **b** old participant

Table 1 presents data from the physiological parameters measured during the two tests for both young and old individuals. In the younger population there was a significantly higher $\dot{V}{\text{O}}_{2\hbox{max} }$ (l min^−1^ and ml kg^−1^ min^−1^), peak $\dot{V}{\text{E}}$, peak RER, peak *Q*, peak SV and peak PO achieved in the SPV compared to the RAMP protocol (*p* < 0.05). The a-vO_2_diff was significantly lower in the SPV compared to the RAMP (*p* < 0.05). There were no differences in AT, peak HR, end-exercise lactate and TTE (*p* > 0.05). Significant differences in peak RER, peak PO and TTE were found between protocols in the older population (*p* < 0.05). Figure 1 shows the $\dot{V}{\text{O}}_{2}$ response over time from a representative participant of each group in both SPV and RAMP protocols. Participants were selected as they were representative of both mean $\dot{V}{\text{O}}_{2\hbox{max} }$ values, and average differences between the two test protocols.

**Table 1:** Measured physiological variables recorded during SPV and RAMP $\dot{V}{\text{O}}_{2\hbox{max} }$ tests for both young and old populations

	Young (18–30 years)			Older (50–75 years)		
	Ramp	SPV	*p* value	Ramp	SPV	*p* value
$\dot{V}{\text{O}}_{2\hbox{max} }$ (l min^−1^)	3.34 ± 0.88	3.45 ± 0.87*	0.02	2.74 ± 0.76	2.78 ± 0.74	0.79
$\dot{V}{\text{O}}_{2\hbox{max} }$ (ml kg^−1^ min^−1^)	47.70 ± 9.98	49.68 ± 10.26*	<0.01	38.99 ± 9.54	39.12 ± 8.61	0.84
AT (ml kg^−1^ min^−1^)	25.36 ± 6.71	24.55 ± 5.18	0.32	21.69 ± 6.17	22.34 ± 6.36	0.23
HR (bpm)	181 ± 10	183 ± 9	0.19	164 ± 12	164 ± 12	0.93
$\dot{V}{\text{E}}$ (l min^−1^)	130.7 ± 32.9	147.7 ± 37.4*	<0.01	122.8 ± 31.4	129.4 ± 29.6	0.08
RER	1.24 ± 0.05	1.31 ± 0.08*	<0.01	1.22 ± 0.09	1.32 ± 0.12*	<0.01
Peak *Q* (l/min^−1^)	23.4 ± 5.9	27.3 ± 3.8*	0.01	24.1 ± 5.0	25.8 ± 5.1	0.24
Peak SV (ml)	134.6 ± 37.5	160.0 ± 27.8*	<0.01	156.9 ± 29.9	166.8 ± 29.0	0.23
End-exercise lactate (mmol/l)	8.06 ± 1.74	9.52 ± 2.85	0.06	6.15 ± 1.88	7.21 ± 2.89	0.05
Peak PO (W)	265 ± 69	336 ± 122*	<0.01	226 ± 63	245 ± 74*	<0.01
a-vO_2_diff (ml 100 ml^−1^)	18.1 ± 5.9	15.5 ± 3.9*	0.04	13.3 ± 2.4	13.9 ± 5.4	0.61
TTE (s)	637 ± 153	600 ± 0	0.26	695 ± 149	600 ± 0*	<0.01

* Significantly different from the ramp (<0.05). Data are presented as mean ± SD

### NIRS

**Fig. 2:**
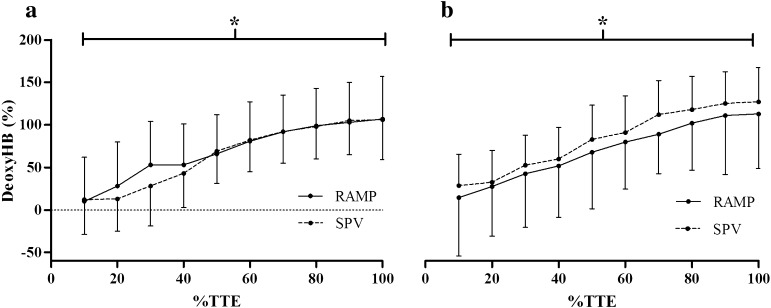
Relative change in deoxyHb vs. %TTE in the RAMP and SPV for: **a** young and **b** old population. *Main effect (*p* < 0.05). Data are mean ± SD

Prior to analysis, data from three participants in the young, and six in the older group were excluded due to a poor quality NIRS signal. There was no interaction effect between test protocol and the increase in deoxyHb over time (*p* > 0.05). Figure 2 illustrates the relative change in deoxyHb over the duration of RAMP and SPV protocols, in the both young and old groups. A main effect of time was evident as deoxyHb increased significantly during both test protocols in both age groups (*p* < 0.01). Specifically, data demonstrated that in the young group, deoxyHb in the RAMP significantly increased up to 70% of TTE (*p* < 0.05); in the SPV there were differences between 30–40, 40–50, and 60–70% of TTE (*p* < 005). For the old group in the RAMP there were differences between 20–30, 30–40, and 40–50% of TTE (*p* < 0.05); in the SPV there were differences between 30–40, 40–50, 50–60, and 60–70% of TTE (*p* < 0.05).

### EMG

**Fig. 3:**
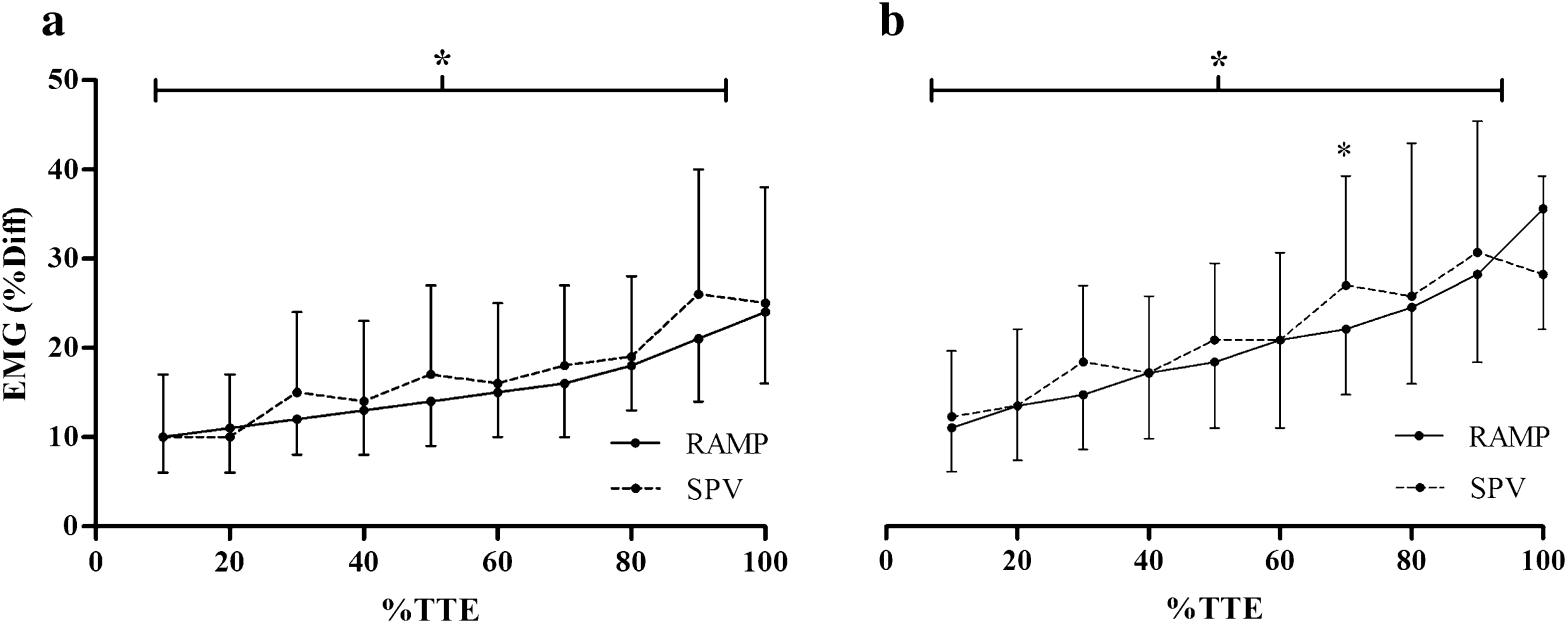
Normalized EMG relative to %TTE over the RAMP and SPV for: **a** young and **b** old populations. *Main effect (*p* < 0.05). Data are mean ± SD

There was no interaction effect evident between the increase of relative EMG between the protocols in the young group. However, in the old population an interaction effect was evident, with post hoc testing demonstrating a significantly higher EMG at 70% of TTE in the SPV compared to the RAMP (*p* < 0.05). There was a main effect of time in both age groups as the relative EMG increased significantly in relation to %TTE (*p* < 0.01; Fig. 3). Post hoc analysis demonstrated that for the young group, there was a significant difference between 80 and 90% of TTE for the RAMP, and between 20 and 30% TTE for the SPV. For the old group, post hoc analysis demonstrated a significant difference between 90 and 100% of TTE from the RAMP, with no significant differences between the successive stages in the SPV. There were no differences in the percentage of change from pre- to post-MIVC EMG or torque between the SPV and RAMP (*p* > 0.05).

### Cardiac Output and Stroke Volume

**Fig. 4:**
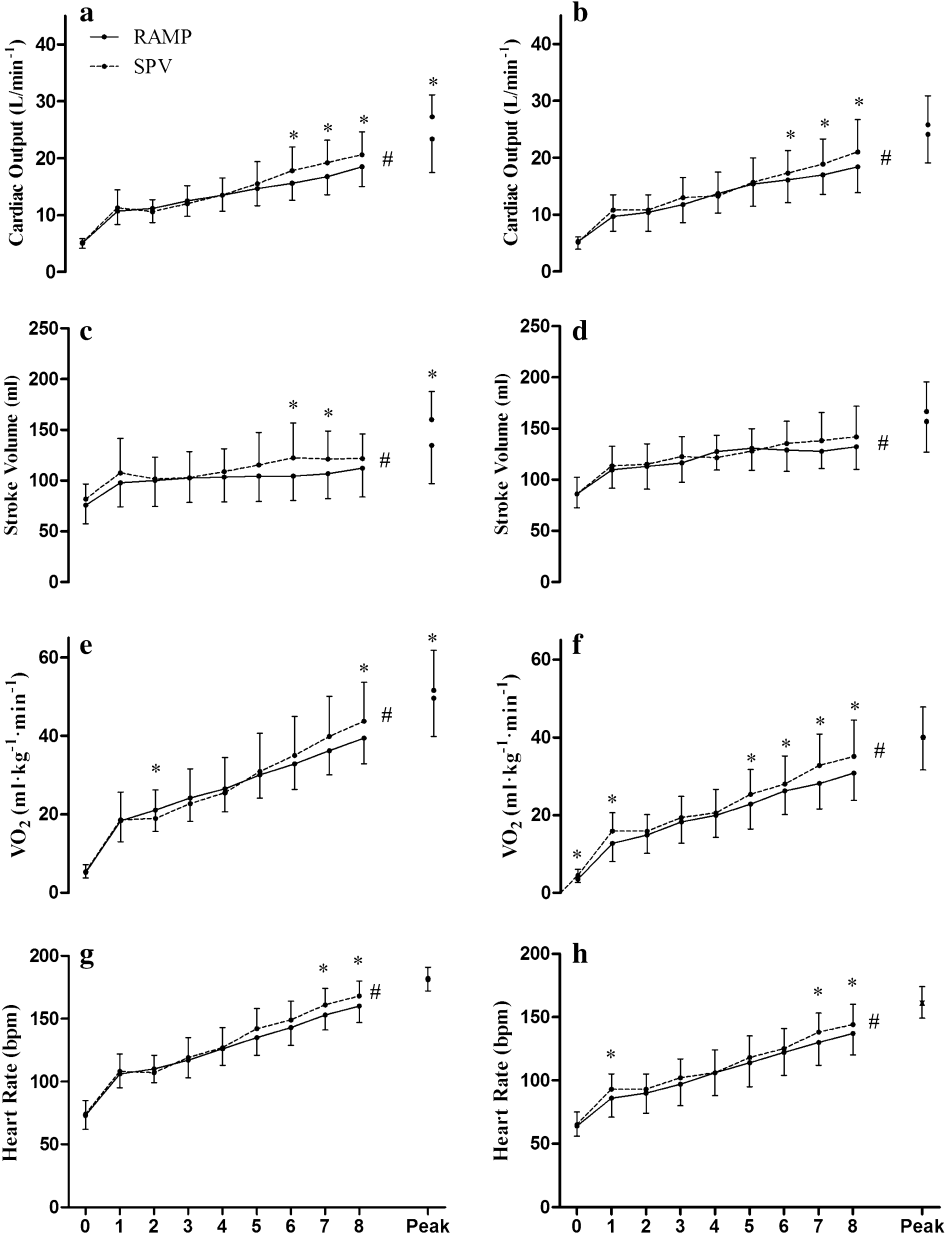
Mean *Q*, SV, $\dot{V}{\text{O}}_{2}$ and HR in both SPV and RAMP protocols in young (*left*; **a**, **c**, **e**, **g**) and old (*right*; **b**, **d**, **f**, **h**) groups. Due to variations in test time from the RAMP protocol values are presented to isotime (8 min), followed by the peak values obtained in each test. ^#^Significant main effect of time (*p* < 0.05). *Significant difference between the SPV and RAMP (*p* < 0.05). Data are mean ± SD

Prior to analysis, data from five participants in the old group (total *n* = 17) and four from the young group (total *n* = 18) were excluded due to a poor quality signal. Figure 4 presents the mean and SD for *Q*, SV, $\dot{V}{\text{O}}_{2}$ and HR as a time series plot. Only those participants with complete data sets were included in this figure, therefore mean values for $\dot{V}{\text{O}}_{2\hbox{max} }$ and HR differ to those presented in the Table 1 where all participants were included. In the young group, there was an interaction effect evident between test protocol and both *Q* and SV over time. Specifically, post hoc testing demonstrated differences in *Q* between the two tests at 6, 7, 8 min as well as the peak value, and SV at 6 min, 7 min, and the peak values (*p* < 0.05). There was a main effect of time (*p* < 0.01), for both *Q* and SV in the young group. Post hoc testing identified significant differences in *Q* occurred between baseline and 1 min, 2 min to 5 min, and 8 min and peak in the RAMP, and between baseline and 1 min, 3 min to 4 min, 5 min to 6 min, 8 min and peak in the SPV. Differences in SV were evident between baseline and 1 min, and 8 min and peak in the both test protocols in the young group (*p* < 0.05). In the old population there was a trend for an interaction effect between protocol and time for *Q* (*p* > 0.07), and a significant interaction for SV (*p* < 0.05). There was main effect for test protocol in both *Q* and SV (*p* > 0.05). However, there was an overall main effect of time for *Q* and SV (*p* < 0.01). Post hoc testing demonstrated differences in *Q* from baseline to 1 min, 2 min to 5 min, and between 8 min and peak in the RAMP, and differences from baseline to 1 min, 2 min to 3 min, and 4 min to 5 min in the SPV. For SV differences were evident from baseline to 1 min, 3 min to 4 min, and 8 min to peak in the RAMP, and differences from baseline and 1 min, and 8 min to peak in the SPV (*p* < 0.05).

### Power Output Profile

**Fig. 5:**
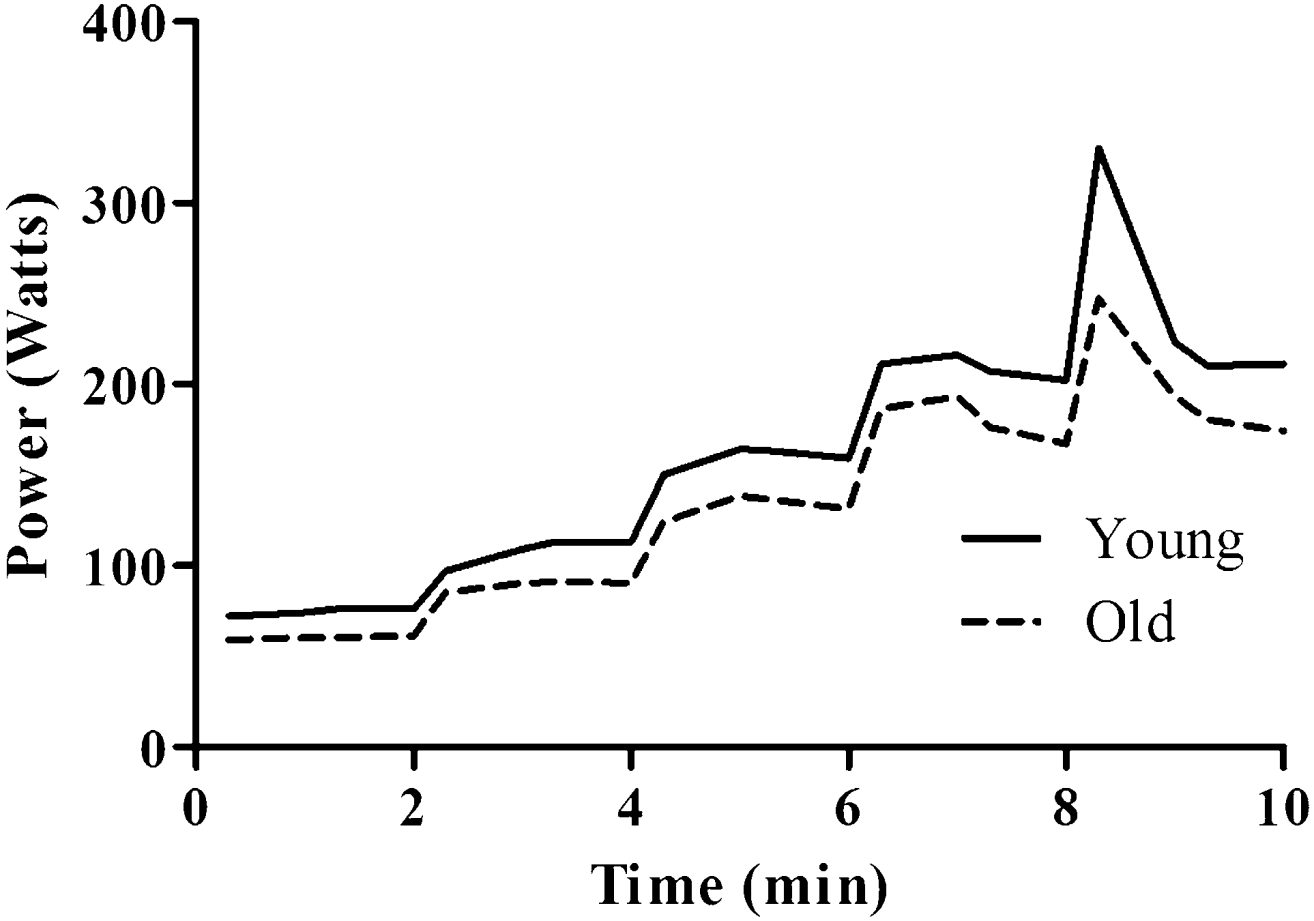
Power output profile from the SPV in the young and old groups

Figure 5 illustrates the mean PO values for each stage of the SPV for both the young and old groups.

## Discussion

This is the first study to assess both the cardiovascular and muscular response to an incremental self-paced exercise test compared to a standard RAMP protocol, and do so in both young and older populations. In support of previous literature (Astorino et al. 2015; Mauger and Sculthorpe 2012; Mauger et al. 2013), the results of the current study demonstrate that younger participants were able to achieve a significantly higher $\dot{V}{\text{O}}_{2\hbox{max} }$ in the SPV compared to the RAMP protocol. However, this was not evident in the older population where there were no differences in $\dot{V}{\text{O}}_{2\hbox{max} }$ between the two protocols. The results also demonstrate that the SPV produced a higher peak *Q* and peak SV in the young group, which was statistically different between the SPV and RAMP at the latter stages of the test. No differences in these parameters were evident between tests in the older group. However, interestingly higher physiological work rates were achieved by participants from both age groups (as evidenced by the significantly greater peak PO and RER values) in the SPV protocol, but this only lead to a higher $\dot{V}{\text{O}}_{2\hbox{max} }$ in the young group. In turn this suggests that the limiting factor to $\dot{V}{\text{O}}_{2\hbox{max} }$ and the mechanisms behind the SPV may differ between young and old populations. Results from the current study also demonstrate a lack of protocol specific patterns of deoxyHb and muscle recruitment of the VL, suggesting that oxygen extraction was not enhanced by the SPV.

In support of findings of the current study, Astorino et al. (2015) demonstrate a significantly higher *Q* and $\dot{V}{\text{O}}_{2\hbox{max} }$ in the SPV when compared against a standard exercise test protocol. The authors suggested that the higher *Q* during the SPV is responsible for the higher $\dot{V}{\text{O}}_{2\hbox{max} }$ observed due to an increased oxygen delivery to the working muscles. It is well accepted that there is a strong linear relationship between *Q* and $\dot{V}{\text{O}}_{2}$, with *Q* being a principal limiting factor for $\dot{V}{\text{O}}_{2\hbox{max} }$ during whole-body exercise (Basset and Howley 2000). Therefore, it would be expected that a higher *Q* would result in a greater $\dot{V}{\text{O}}_{2\hbox{max} }$ being achieved. Results from the current study demonstrate a significantly higher *Q* achieved in the SPV vs. the RAMP in the young, but not the old population. Interestingly, with $\dot{V}{\text{O}}_{2\hbox{max} }$ only being significantly higher in SPV in the young group, it suggests that *Q* might be the primary limiter. However, it must be acknowledged that peak values for *Q* and $\dot{V}{\text{O}}_{2\hbox{max} }$ may not have necessarily occurred at the same time in the SPV, and so caution must be applied when identifying an increased *Q* as the sole explanation for the higher $\dot{V}{\text{O}}_{2\hbox{max} }$ on the basis of these data. Nevertheless, the same maximal heart rate achieved in both protocols suggests that the enhanced *Q* seen in the young group is predominantly the result of the higher SV achieved in the SPV vs. RAMP. However, as stated above, this cannot be confirmed as peak SV and peak HR may not have necessarily occurred at the same time point. Indeed, previous literature has suggested that differences in $\dot{V}{\text{O}}_{2\hbox{max} }$ between individuals are primarily a result of the differences in maximal SV, as less inter-individual variation is seen in maximal HR (Basset and Howley 2000). In traditional $\dot{V}{\text{O}}_{2\hbox{max} }$ tests it is known that SV begins to plateau/fall prior to $\dot{V}{\text{O}}_{2\hbox{max} }$ being reached, whilst HR continues to increase to a maximal level, thus causing a plateau in *Q* near the end of the exercise test (Mortensen et al. 2005). This demonstrates an impairment of the circulatory system to continue supplying a linear increase in oxygen delivery at higher exercise intensities (Mortensen et al. 2005). The main cause for the plateau/fall in SV is suggested to be predominately attributed to a decrease in diastolic filling time as a result of the increasing heart rate (Higginbotham et al. 1986; Vella and Robergs 2005). From the data presented in Fig. 4 it is evident that SV in the young group increases to a greater extent in the SPV than in the RAMP. A plateau in SV is also evident where the rate of increase from the early stages of the test appears to “level-off” sooner in the RAMP than in the SPV. As a result, the increase in *Q* during the final stage of the SPV test is greater than in the RAMP protocol. In the older group a plateau in SV is evident in both tests, although there is a trend for SV to be higher in the latter stages of the SPV compared to the RAMP. This, combined with the significantly higher HR in minute 7 and 8 during the SPV, may partly explain the observed higher *Q* in the 6th, 7th and 8th minute.

As participants are free to make adjustments in work rate, it may be that the self-paced nature of the SPV (particularly in the younger populations), contributes to the prevention of an early plateau in SV by potentially creating more optimal physiological conditions to maintain adequate oxygen delivery. However, further research is required to test this hypothesis and examine the underpinning mechanisms behind the SV response. Despite the young group’s higher peak *Q*, their peak a-vO_2_diff was lower in the SPV, which would have potentially negatively affected the magnitude of the observed $\dot{V}{\text{O}}_{2\hbox{max} }$. Unfortunately, data from the current study cannot identify reasons for the observed lower a-vO_2_diff, but an oxygen diffusion limitation (caused by the higher *Q*), or reductions in muscle blood flow may be likely candidates. Future studies using invasive, direct measures of a-vO_2_diff are necessary to investigate these proposed mechanisms.

Older participants demonstrated no significant differences in $\dot{V}{\text{O}}_{2\hbox{max} }$ between the SPV and RAMP protocols. It is possible that the effect of the protocol is reduced in the older group due to their inability to further increase *Q* in response to the increased work rates that the SPV protocol allows (as seen in the young group). Indeed, previous research has suggested that *Q* plateaus at around ~80% of peak PO (Mortensen et al. 2005), and even though in the young group there were no significant differences in the increase of *Q* between the final time points (6–8 min) of the SPV (*p* > 0.05), the pattern of response is not the same as in the RAMP (see Fig. 4). Moreover, even though there were no differences in peak *Q* in the old group between the two tests, they still achieved a higher PO in the SPV vs. the RAMP. It is not clear why this differential response in *Q* has been observed between age groups across the test protocols, but it has been suggested that there are age-related changes which occur in relation to cardiac function in healthy individuals. In particular these changes in cardiac function include left ventricular wall thickness, reductions in diastolic filling, impaired left ventricular ejection fraction, and reductions in HR (Lakatta and Levy 2003). All of these changes are known to influence cardiac function (Lakatta and Levy 2003). In particular, reductions in diastolic filling time is suggested to be the primary cause of the plateau that occurs in SV above a certain exercise level in older individuals (Higginbotham et al. 1986; Vella and Robergs 2005; Lakatta and Levy 2003). This could be the key reason why the older group did not achieve a higher peak *Q* and therefore $\dot{V}{\text{O}}_{2\hbox{max} }$ during the SPV (as seen in the young group). However, interestingly, higher absolute peak *Q* and SV values were found in the older group compared to the young group in both test protocols, which suggests reduced cardiac function was not evident in this older population.

A further speculative reason for the divergent protocol effects on $\dot{V}{\text{O}}_{2\hbox{max} }$ between the age groups could be due to the known age-related changes that occur at the periphery. Research has suggested that there is a reduced muscle oxidative capacity in older populations (Betik and Hepple 2008; Russ and Kent-Braun 2004) due to loss in mitochondrial content and function, and a reduction of muscle volume (Conley et al. 2000). This reduced oxidative capacity is likely to affect the a-vO_2_diff, which according to the Fick equation, contributes to the attainment of $\dot{V}{\text{O}}_{2\hbox{max} }$. In support of this speculation, data from this study demonstrate that the a-vO_2_diff is lower in the old compared to the young group. A further possibility is that due to the reduced lung performance associated with normal age-related decline (Chaunchaiyakul et al. 2004; Janssens et al. 1999), there is an increase in the oxygen cost of breathing meaning that more *Q* is needed to be directed to the lungs to support ventilation during exercise (Proctor et al. 1998). Indeed, a 20–30% decrease in leg blood flow during cycling has been shown in older, compared to younger subjects (Proctor et al. 1998). Thus, age-related reductions in leg muscle blood flow may have affected oxygen delivery to the working muscle and consequently limited a-vO_2_diff and $\dot{V}{\text{O}}_{2\hbox{max} }$.

Previous literature has shown the SPV to produce higher $\dot{V}{\text{E}}$ values when compared to a standard $\dot{V}{\text{O}}_{2\hbox{max} }$ protocol in a young population (Astorino et al. 2015; Faulkner et al. 2015; Hogg et al. 2014; Mauger et al. 2013). Interestingly, studies that demonstrate no difference in $\dot{V}{\text{O}}_{2\hbox{max} }$ between the SPV and standard RAMP protocols also failed to find differences in $\dot{V}{\text{E}}$ (Chidnok et al. 2013; Straub et al. 2014). The greater $\dot{V}{\text{E}}$ demonstrated by younger participants in the current study, and also reported in previous studies (Astorino et al. 2015; Faulkner et al. 2015; Hogg et al. 2014; Mauger et al. 2013), is likely due to the “all-out” effort required during the final stage (RPE 20) of the test. End-test lactate values (see Table 1) suggest that this “all-out” effort results in a greater level of metabolic stress than experienced in standard RAMP testing. The greater level of acidosis and metabolic buffering would therefore increase the ventilatory response during high intensity exercise (Milani et al. 2006). However, Mauger and Sculthorpe (2012) observed no differences in $\dot{V}{\text{E}}$ between the RAMP and SPV protocols even though a higher $\dot{V}{\text{O}}_{2\hbox{max} }$ was achieved in the SPV. Therefore, it is still questionable whether or not the higher $\dot{V}{\text{O}}_{2\hbox{max} }$ resulting from the SPV protocol is a result of a higher rate of $\dot{V}{\text{E}}$.

Interestingly, in contrast to the current findings, Chidnok et al. (2013) did not find a higher $\dot{V}{\text{O}}_{2\hbox{max} }$, $\dot{V}{\text{E}}$ or end-test blood lactate concentration from a SPV protocol compared to a standard RAMP test. However, the differences in the outcomes between Chidnok et al. and the current study could be attributed to the SPV test protocol designs. As outlined above, the SPV protocol from the current study requires an “all-out” effort to be maintained for the duration of the final stage. This “all-out” maximal effort is very different to pacing a maximal effort (RPE 20) as the highest work rate that can be sustained for the duration of the stage, as required by the protocol of Chidnok et al. (2013). This fundamental difference between the two test protocols is the likely reason for the disparity between the findings of the two studies. Indeed, data from the current study demonstrate a mean decrease in PO in the final stage of 120 W for the young group, and 73 W for the older group. This is in contrast to the data from Chidnok’s study which demonstrate a mean reduction in PO of just 20 W during the final stage of their SPV protocol. Thus, it could be suggested the final stage “all-out” effort might be required in order to drive the mechanisms that pertain to the higher $\dot{V}{\text{O}}_{2\hbox{max} }$ in SPV tests using this protocol design. Interestingly, the PO values at the point of $\dot{V}{\text{O}}_{2\hbox{max} }$ in the current study were significantly higher in the RAMP compared to the SPV in both the young (RAMP 263 W; SPV 219 W; *p* < 0.01), and old group (RAMP 222 W; SPV 195 W; *p* < 0.01). These findings suggest that in the SPV, $\dot{V}{\text{O}}_{2\hbox{max} }$ values seem to occur when PO is submaximal. Previous research supports this finding and has demonstrated that PO can be dissociated with $\dot{V}{\text{O}}_{2\hbox{max} }$; i.e. PO does not necessarily have to be maximal to achieve maximal $\dot{V}{\text{O}}_{2}$ values (Billat et al. 2013, Milani et al. 2006). Thus, the nature of the “all-out” final stage of the SPV test (RPE 20), and time delay in the oxygen uptake kinetic response, is likely to be the reason for the dissociation between $\dot{V}{\text{O}}_{2\hbox{max} }$ and the peak PO values.

## Limitations

A limitation of the current study is that different cycle ergometers were used to complete the RAMP and SPV. It has previously been suggested that different ergometers might cause differences in metabolic cost and cardiovascular strain (Reiser et al. 2000). Indeed, various factors such as saddle angle (Umberger et al. 1998), body positioning (Too 1991) and saddle to pedal distance (Too 1993) have been shown to influence maximal cycling performance. However, different ergometers were necessary to be able to conduct the two protocols as the SPV required participants to freely adjust their PO, and the RAMP required accurate fixing of PO. Even though different ergometers were used, the current study demonstrated similar magnitude of differences in $\dot{V}{\text{O}}_{2\hbox{max} }$ between RAMP and SPV as those reported in previous studies where the same ergometer was used for both tests (Astorino et al. 2015, Mauger and Sculthorpe 2012). Chidnok et al. (2013) also used different cycle ergometers when making comparisons between the SPV and standard RAMP protocols but found no significant differences in $\dot{V}{\text{O}}_{2\hbox{max} }$.

The use of non-invasive techniques to estimate *Q* and SV have previously been criticised for their lack or accuracy and reliability when compared against more invasive techniques (e.g. direct Fick method), with the typical error being reported to be around 9% for peak *Q* and SV (Welsman et al. 2005). There is also the possibility that movement and respiratory artefacts associated with exercise may have affected our results (Siebenmann et al. 2015). However, previous research has demonstrated that non-invasive devices such as that used in the current study are a reliable and provide clinically acceptable measures of *Q* and SV in adults during exercise (Charloux et al. 2000, Richard et al. 2001). Nevertheless, the authors accept that caution must be applied when drawing conclusions from such measures.

The SPV allows participants to freely adjust both workload and cadence. Previous research by Gottshall and colleagues (Gottshall et al. 1996) has suggested that large variations in cadence could influence the muscle blood flow and *Q* response to exercise. Thus any differences in cadence between SPV and RAMP protocols may have influenced the current results. However, the study by Gottshall et al. (1996) was completed during submaximal steady state exercise, and we are unaware of any studies that have presented similar data obtained during maximal incremental exercise. Therefore, it is difficult to determine whether cadence differences were a confounding variable within our study. Nevertheless, the mean cadence from both test protocols were similar in the young (RAMP 76 rev min^−1^; SPV 78 rev min^−1^), and identical in the old group (77 rev min^−1^). We are therefore confident that cadence is unlikely to have had a substantial influence on the results.

## Conclusion

In agreement with the findings of previous research, the current study demonstrates that the SPV produces higher $\dot{V}{\text{O}}_{2\hbox{max} }$ values compared to a standard RAMP based protocol in a young healthy population. It is possible that the higher $\dot{V}{\text{O}}_{2\hbox{max} }$ in the SPV protocol is the result of an increase in the oxygen delivery (increased *Q* and $\dot{V}{\text{E}}$), rather than a result of any increase or change in the oxygen extraction at a muscular level. However, it is unclear if these peak values all occurred at the same time, and therefore whether they mechanistically explain the higher $\dot{V}{\text{O}}_{2\hbox{max} }$ seen in the SPV protocol. In contrast, no differences in $\dot{V}{\text{O}}_{2\hbox{max} }$ between the SPV and RAMP protocols were seen in the older population, despite greater peak RER and PO values being achieved in the SPV. The reasons for the divergent findings between age groups are unclear, although age-related changes in the physiological response to exercise are a possible explanation. Nonetheless, these findings suggest that the SPV protocol enables participants achieve work rates that are closer to true physiological maximum, compared to a traditional RAMP protocol. Whilst this is manifested as a higher $\dot{V}{\text{O}}_{2\hbox{max} }$ in young populations, a similar $\dot{V}{\text{O}}_{2\hbox{max} }$ is observed in older populations. Therefore, this study demonstrates that the SPV is a valid determinant of $\dot{V}{\text{O}}_{2\hbox{max} }$ in both young and old populations, and provides a more adequate assessment of a participant’s maximal aerobic capacity.

## Abbreviations

$\dot{V}{\text{O}}_{2\hbox{max} }$: Maximal oxygen update
SPV: Self-paced $\dot{V}{\text{O}}_{2\hbox{max} }$ test
RPE: Ratings of perceived exertion
HR: Heart rate
VE: Minute ventilation
*Q*: Cardiac output
SV: Stroke volume
PO: Power output
AT: Anaerobic threshold
RER: Respiratory exchange ratio
DeoxyHb: Muscle deoxyhaemoglobin
NIRS: Near-infrared spectroscopy
VL: Vastus lateralis
EMG: Electromyography
%TTE: Percentage of time to exhaustion
MIVC: Maximal isometric voluntary contraction
